# Sensitivity of migratory connectivity estimates to spatial sampling design

**DOI:** 10.1186/s40462-021-00254-w

**Published:** 2021-04-02

**Authors:** Stephen H. Vickers, Aldina M. A. Franco, James J. Gilroy

**Affiliations:** School of Environmental Sciences, University of East Anglia, Norwich, NR4 7TJ UK

**Keywords:** Mantel test, Migratory connectivity, Migration, Migratory spread, Sampling bias

## Abstract

**Background:**

The use of statistical methods to quantify the strength of migratory connectivity is commonplace. However, little attention has been given to their sensitivity to spatial sampling designs and scales of inference.

**Methods:**

We examine sources of bias and imprecision in the most widely used methodology, Mantel correlations, under a range of plausible sampling regimes using simulated migratory populations.

**Results:**

As Mantel correlations depend fundamentally on the spatial scale and configuration of sampling, unbiased inferences about population-scale connectivity can only be made under certain sampling regimes. Within a contiguous population, samples drawn from smaller spatial subsets of the range generate lower connectivity metrics than samples drawn from the range as a whole, even when the underlying migratory ecology of the population is constant across the population. Random sampling of individuals from contiguous subsets of species ranges can therefore underestimate population-scale connectivity. Where multiple discrete sampling sites are used, by contrast, overestimation of connectivity can arise due to samples being biased towards larger between-individual pairwise distances in the seasonal range where sampling occurs (typically breeding). Severity of all biases was greater for populations with lower levels of true connectivity. When plausible sampling regimes were applied to realistic simulated populations, accuracy of connectivity measures was maximised by increasing the number of discrete sampling sites and ensuring an even spread of sites across the full range.

**Conclusions:**

These results suggest strong potential for bias and imprecision when making quantitative inferences about migratory connectivity using Mantel statistics. Researchers wishing to apply these methods should limit inference to the spatial extent of their sampling, maximise their number of sampling sites, and avoid drawing strong conclusions based on small sample sizes.

**Supplementary Information:**

The online version contains supplementary material available at 10.1186/s40462-021-00254-w.

## Introduction

Understanding animal migration – the cyclical movements of individuals between areas used across the annual cycle – is challenging, yet is often a prerequisite for effective conservation of mobile species. Our capacity to measure migratory movements has improved greatly in recent years through direct methods such as mark-recapture [1] and remote-tracking technology [2], as well as indirect methods such as genetic [3] and biogeochemical approaches [4]. With an improved understanding of individual migratory movements, researchers are increasingly focussing on quantifying resultant population-level spatial patterns. Understanding migratory connectivity (henceforth referred to as ‘connectivity’), which describes the extent to which spatial distributions of individuals are maintained between two phases of the migratory cycle (most often between breeding and non-breeding grounds), has become a top priority [5]. High levels of connectivity indicate that individuals residing close together in a particular season of the annual cycle are also close together in a subsequent season, whilst low connectivity indicates cross-seasonal mixing of individuals from different areas. The strength of connectivity can have important conservation implications, including playing a key role in a migratory species’ propensity to adapt to a changing environment [6, 7].

Multiple statistical approaches to estimate migratory connectivity have been utilised in recent years [5, 8, 9]. To quantify the strength of connectivity (i.e. giving connectivity a numerical value) one of the most commonly used approaches is the Mantel test [10], which evaluates the correlation between two distance matrices: the pairwise distances between locations of sampled individuals in one season, and their equivalent pairwise distances in another [11]. Numerous studies have examined sources of bias in connectivity estimates derived using Mantel correlations, including issues of imbalanced sampling with respect to local abundance, incomplete spatial coverage, and location uncertainty [11, 12]. An extension to the Mantel approach [12] utilises the transition rates of individuals between pre-defined breeding and non-breeding zones to control for these biases, but this method is only recommended in situations where spatial subpopulation structure is well understood, and relative abundances within origin regions can be estimated. Cohen et al. [12] recommend using Mantel correlations when these conditions are not met, and the Mantel approach remains widely used in recent literature (e.g. [13–16], but see [9, 17]).

One issue that has received little attention in the migratory connectivity literature is the extent to which Mantel correlations can be used to draw inferences about connectivity patterns at the population scale, given that these correlations show scale-dependence [18, 19]. A key aim of migratory connectivity research is to understand migratory patterns at large spatial scales (e.g. whole species ranges), requiring an implicit assumption that metrics quantified for sampled individuals accurately describe behaviour of wider populations. However, these broad-scale inferences have the potential to be strongly biased in some cases, as a product of fundamental sensitivity of connectivity metrics to the spatial extent and configuration of sampling. Estimates also frequently suffer from low precision due to sample size constraints, as the number of individuals tracked within a population is often limited by available funding resources, difficulty in retrieving tracking devices, fieldwork limitations related to catching individuals, site fidelity, and recapture rates [12]. These limitations reduce the proportion of the population that is actually studied, and mean relatively small sample sizes are commonplace in remote-tracking studies [20]. Whilst lower precision can be partially accounted for through bootstrapped confidence intervals, the extent to which precision varies with sample has not been explored in detail [12].

Here, we use simulations to elucidate the direct mechanisms underpinning bias and imprecision in migratory connectivity estimates that use Mantel statistics. We examine the efficacy of multiple sampling scenarios across a range of connectivity levels, considering both homogenous and spatially-clumped populations. We test how the number of individuals sampled impacts the precision of measurements, and examine how the magnitude of bias depends on the extent to which estimates from sampled individuals are used to draw inferences about the wider populations from which they are drawn. Alongside simple generalised simulations that allow us to explore underlying mechanisms of bias, we also use more realistic simulated migratory populations to provide recommendations on study design that can maximise the accuracy of Mantel-based connectivity measures, within realistic limits of sampling.

### Mechanisms of bias

**Fig. 1:**
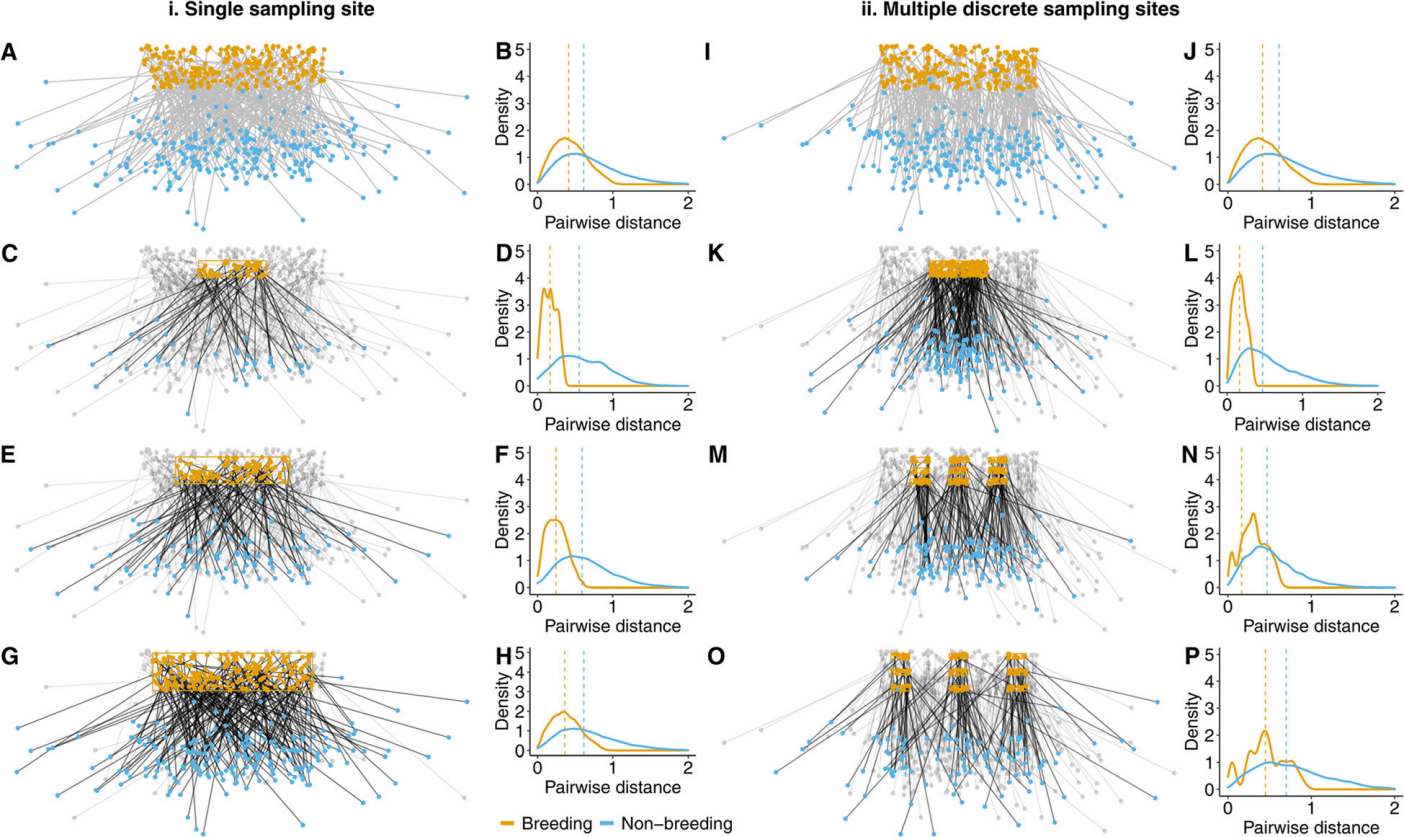
Hypothetical examples of spatial sampling impacts on season-specific pairwise distances between individuals, considering scenarios where sampling occurs within single regions (**a-h**) and discrete sites (**i-p**). Panels **a** and **i** show the spatial distributions of all breeding (yellow dots) and non-breeding locations (blue dots) for two simulated migratory populations, while panels **b** and **j** show the corresponding frequency distributions of pairwise distances between individuals during breeding (yellow line) and non-breeding (blue line) seasons. Panels **c-h** illustrate how sampling (yellow box) individuals across increasingly large spatial subsections the total population influences the observed distributions of breeding pairwise distances, while winter pairwise distances remain relatively unchanged. Panels **k-p** show corresponding scenarios with sampling limited to discrete sites that vary in their spread across the breeding range, with similar impacts on observed breeding pairwise distances

To illustrate the fundamental issue arising from spatial sampling bias, we first consider two hypothetical sampling scenarios for a contiguous breeding population with low connectivity (Fig. 1): one where individuals are marked randomly within a single study region of varying size (Fig. 1a-h) and another where individuals are marked within discrete sampling sites that are spread across the range (Fig. 1i-p). In both cases, the plausible range of observable distances between marked individuals is constrained by sampling extent in the season that marking takes place, which is the breeding range in our hypothetical scenario (see Fig. 1). Importantly, however, the maximum measurable distance between these sampled individuals in the non-breeding range is not constrained by sampling design, only by the destinations of the animals themselves. This could introduce a skew in the sample of pairwise distances on the sampled range (breeding grounds in this case), but not on the destination range (non-breeding grounds). As Mantel correlations explicitly compare these pairwise distance distributions between seasons, resulting Mantel statistics calculated for spatially-constrained samples may be very different from the ‘true’ values calculated for the whole population, despite the underlying migratory ecology being constant across the population (as in Fig. 1).

Biases resulting from spatially-constrained sampling regimes could take various forms, depending on how sampling effort is distributed across the species’ range. If sampling is limited to a subset of the breeding distribution (e.g. Fig. 1c), the observed distribution of breeding pairwise distances will be left-skewed relative to the true distribution across the population (Fig. 1d), leading to negative bias in Mantel correlations with respect to true statistic for the wider population. If sampling occurs in discrete areas that are widely separated across the range of a species, however, resulting pairwise distances may be right-skewed relative to the population as a whole (e.g. Fig. 1p) because site spacing introduces abrupt artificial gaps into what may be a more uniform underlying distribution of individuals across space. Given inevitable logistical constraints, migration studies do indeed typically focus on marking individuals at discrete sites within spatially-constrained study areas [18, 21–23], with considerable variation in the extent to which these are spread across full ranges. This suggests there may be constraints on the extent to which such studies can draw inferences about the connectivity of wider populations using Mantel statistics calculated from spatially-constrained samples. In the next sections, we use simulations to estimate the severity of these biases under a range of common sampling scenarios.

### Simulation methods

To examine fundamental sources of bias, we first simulated simple migratory populations that vary in their degree of migratory connectivity (Fig. A1), and applied a range of sampling regimes to examine how Mantel statistics resulting from realistic simulated ‘studies’ compared to ‘true’ values calculated for the simulated population as a whole.

#### Simulating breeding and non-breeding locations

First, we created a breeding range filled with *N* individuals, placed at random by sampling x and y coordinates from a bounded uniform distribution ([24], Fig. A1A, *N* = 10,000), ensuring that variation in our results reflected sampling effects alone, rather than stochasticity arising from heterogeneous spacing of individuals. We simulated migratory movements by 1) shifting each individual a fixed distance due south from its breeding location (Fig. A1A), and then 2) further shifting the individual in a random direction (sampled from a uniform distribution between 0 and 360; Fig. A1B-C). The distance of this second shift was sampled from a lognormal distribution with SD = 1, and a mean that we varied across scenarios, allowing us to simulate different strengths of connectivity (values of 3, 5 and 7 were used).

#### Simulating study designs

Two basic sampling designs were applied to simulated populations (Fig. 1):

##### Area scenarios

To test for the effect of sampling area size, a single rectangular sampling area was used, centred within the breeding area, within which 200 individuals were sampled at random for tracking. The size of this rectangular area was varied to generate three scenarios of increasing total study area with sample size held constant (sampling areas are illustrated in Fig. 1c, e, & g).

##### Spread scenarios

To test for effects of sampling ‘spread’ under a fixed study area design, 200 individuals were randomly sampled from nine rectangular sampling areas (sites) distributed in a 3 × 3 grid formation centred within the breeding area. Spacing between these sites was then varied to generate three scenarios of differing sampling spread, holding the size of sampled area and sample size constant in each case (sampling sites are illustrated in Fig. 1k, m, & o).

We generated 100 replicate datasets for each scenario (area and spread), and repeated this for each of the three strength levels of connectivity tested.

#### Estimating migratory connectivity

Using sampled individuals from each scenario, we calculated Mantel correlations using the *mantel.rtest* function within the *ade4* package in R [25]. In each case, we assumed that all individuals sampled in the breeding range were tracked successfully to their winter locations and there was no location uncertainty. Scores were then assessed with respect to: 1) the difference between the observed Mantel score and the ‘true’ value calculated for the entire global population of 10,000 individuals, and 2) the difference between the observed Mantel score and an equivalent ‘true’ value calculated using all individuals inhabiting the strict spatial extent of sampling (henceforth ‘zone’).

#### Sample size scenarios

We tested a range of sample sizes to examine how precision varies in relation to the proportion of a population being sampled. For each level of connectivity, we randomly sampled individuals from the entire breeding range (global population *N* = 10,000), applying sample sizes of 10 (0.1%), 50 (0.5%), 100 (1%), 1000 (10%), 2500 (25%), and 5000 (50%) individuals. For each sample size and connectivity scenario, 100 replicates were generated with Mantel scores calculated following the previously described method. Bias was determined as the difference between the observed score and the values for the entire simulated population of 10,000 individuals.

#### Patchy population scenarios

Populations in the real world seldom conform to contiguous blocks, and often show a patchy distribution. To examine how this patchiness influences the effect of spatial sampling design on Mantel statistics, we simulated populations inhabiting four equal-sized sub-populations situated at the corners of the breeding range, within which individuals were distributed at random (Fig. A2). Migrations were then simulated using the same process described above (see Fig. A1), but populations were then further restricted to include only individuals that reach four equal-sized regions in the non-breeding area. This was to ensure clearly delimited sub-populations during both the breeding and non-breeding period. We then applied a rectangular sampling area centred within each breeding sub-population, across which 200 individuals were sampled at random for tracking. The size of the rectangular areas was then varied to generate three scenarios of increasing total study area (with sample size held constant).

#### Simulating realistic species ranges

To examine how spatial sampling designs influence connectivity estimates when applied to more realistic migratory populations, we generated further simulated populations that were constrained within real-world breeding and non-breeding BirdLife range maps [26] for three bird species selected to represent diverse range structures (Henslow’s Sparrow *Passerculus henslowii*, Aquatic Warbler *Acrocephalus paludicola*, and Falcated Duck *Mareca falcata*; note that subsequent simulated populations are not intended to be accurate replications of these species). To simulate realistic distributions of individuals within each range, we applied an algorithm to generate spatially-autocorrelated occurrence patterns (i.e. spatial clustering of individuals rather than a uniform distribution) using the *nlm_gaussianfield* function from the NLMR package [27] to generate a Gaussian random field of spatially-clustered values (scaled to vary between 0 and 1), applying an autocorrelation range of 10 and a magnitude of variation of 100 to generate spatial clustering (Fig. A3A). We then distributed 50,000 individuals across each range in proportion to the resulting random field values (Fig. A3B), with spatial autocorrelation ensuring that individuals were clustered in space, with areas of high and low abundance.

To generate a range of differing levels of migratory connectivity for each simulated species range, we used an algorithm that matched breeding and non-breeding locations for individuals according to their longitudinal ranks (Fig. A3C). For each individual in the breeding range, we randomly-selected a non-breeding location from all available points within a given bandwidth of longitudinal rank, and controlled connectivity levels by varying this bandwidth. For example, with a bandwidth of 1000, an individual of longitudinal rank of 5000 on the breeding zone would be randomly assigned a point from all those between longitudinal ranks 4000—6000 on the non-breeding zone. Larger bandwidths of longitudinal ranking on the non-breeding zone therefore result in lower migratory connectivity. Bandwidth sizes of 1000, 13,000, and 25,000 were used to produce three levels of migratory connectivity for each species. To sample the resulting populations, we assigned discrete study areas of fixed size to the 20 highest-density cells within a coarse grid overlain across the breeding range (Fig. A3D–E). This reflects the common logistical constraints (difficulty in catching individuals for tagging, access restrictions, and financial limitations) that may force researchers to restrict their sampling to areas where their chosen species are known to be more abundant. We then selected 200 individuals at random from these sampling sites. To explore the impact of variation in spatial sampling extent on resulting connectivity estimates, we varied the number of sampling sites from which these individuals were drawn, ranging from 3 to 20 sites selected at random from the pool of 20. We repeated this 100 times for each possible sampling scenario and level of connectivity and calculated resulting Mantel scores as well as the mean distance between centroids of sampling sites.

All simulations and statistical analysis were performed with R 4.3.0 (R [28]). Scripts for the completed analysis, including all simulations, are available as electronic supplementary material.

## Results

### Spatial scale fundamentally affects migratory connectivity scores

**Fig. 2:**
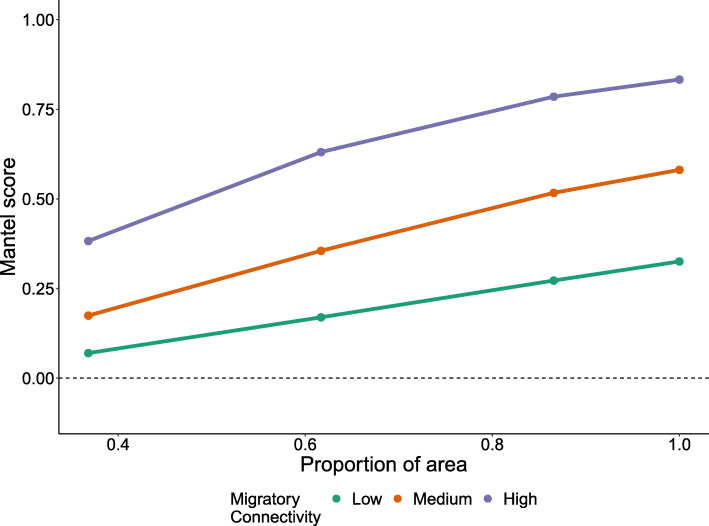
Migratory connectivity scores calculated by the Mantel method for all individuals (i.e. ‘true’ population-scale values) within increasingly-sized spatial zones (rectangular areas centred within the breeding population) within the whole population. Colours represent the three levels of migratory connectivity tested

When calculated for increasingly-sized zones within a single uniformly distributed breeding population, Mantel correlation values calculated for the entire population within the zone always increase (Fig. 2). In effect, the ‘true’ migratory connectivity of a population (measured using all individuals) is dependent upon the absolute size of the area sampling occurs, even when the underlying mechanism generating connectivity for individuals is uniform across the population. This is because the Mantel method fundamentally depends on the relative spatial arrangement of individuals within each zone of interest, and hence are highly sensitive to the spatial characteristics of those zones. The more a given zone is spatially restricted by sampling, the narrower the subset of measurable pairwise distances (Fig. 1). In the case of connectivity studies where individuals are marked within one seasonal range, the censoring of pairwise distances does not take place to the same extent on the other range. This mis-match in censoring of pairwise distances results in lower Mantel scores relative to a population that is less spatially restricted (Fig. 2).

Therefore, inferences drawn from Mantel scores about the migratory connectivity of whole populations may be systematically biased if those scores come from spatially-constrained samples of individuals. When applying area-based sampling regimes (i.e. random sampling across a single zone as shown in Fig. 1i), this spatial sampling artefact resulted in consistent underestimation of connectivity scores relative to ‘true’ values for the whole population. Underestimation was more severe as the disparity between the size of the sampled area and the total area occupied by the population increases. The effect was consistent across the three strengths of connectivity tested, but was strongest for populations with weaker connectivity (Fig. A4).

### Making inferences at the spatial extent of sampling can still be biased

**Fig. 3:**
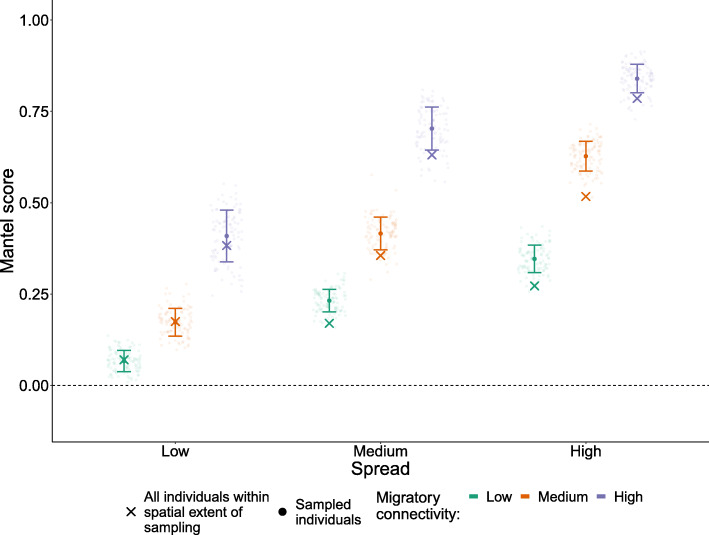
Mantel scores from 100 replicate simulated studies (circles) compared to that of all individuals within the spatial extent of sampling (crosses). Each replicate is calculated using 200 individuals sampled randomly across nine sampling areas which were varied in their spread. Error bars indicate standard deviation around the mean score of 100 replicates

If inferences about the strength of connectivity are made explicitly for the individuals inhabiting the spatial extent of sampling (and not wider populations), area-based sampling scenarios produced Mantel scores without significant bias (Fig. A5). However, the commonly-used scenario of sampling from multiple discrete study areas (‘spread’ scenarios, as shown in Fig. 1ii) lead to overestimation of connectivity in medium and high spread scenarios, even when inference was restricted solely to the populations within the sampled space (Fig. 3). This overestimation results from the large distances between sampling sites, which over-selects individuals of high pairwise distance during the breeding season, leading to a right-skewed sample of pairwise distances relative to the distribution for all individuals within the spatial extent of sampling (Fig. A6).

### Large sample sizes are required to achieve precision

**Fig. 4:**
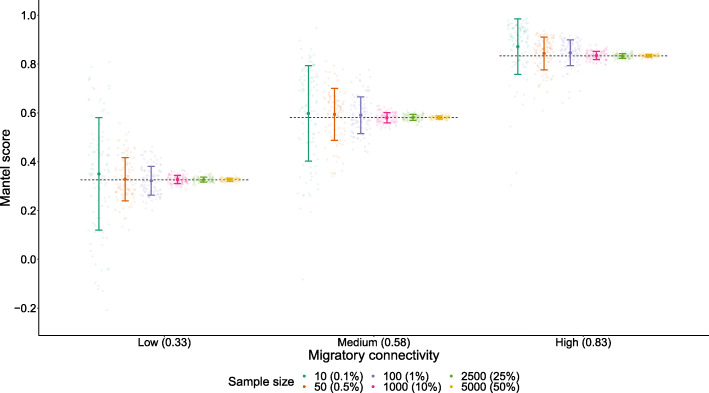
How number of individuals that are sampled influences precision in Mantel scores. Each point represents a replicate simulated study for a given sample size, randomly sampled from across the whole population. Error bars indicate standard deviation around the mean score of 100 replicates

With random sampling across a single contiguous uniform breeding population, we found large variation in connectivity estimates across the 100 replicate simulated studies with sample sizes of 100 or less (Fig. 4). Standard deviations around the mean of connectivity estimates were largest for lower connectivity scenarios, with SD as high as 0.23 (under sample size of 10*,* ‘low’ connectivity). With sample sizes of 1000 and above, SD around mean connectivity estimates fell to a negligible level in the context of a score on the scale of − 1 to + 1 (SD < 0.025 across all strengths of connectivity tested). This indicates that with sample sizes of 100 individuals or fewer, precision of resultant scores may be very low even under ideal sampling designs.

### Patchy populations also show bias

**Fig. 5:**
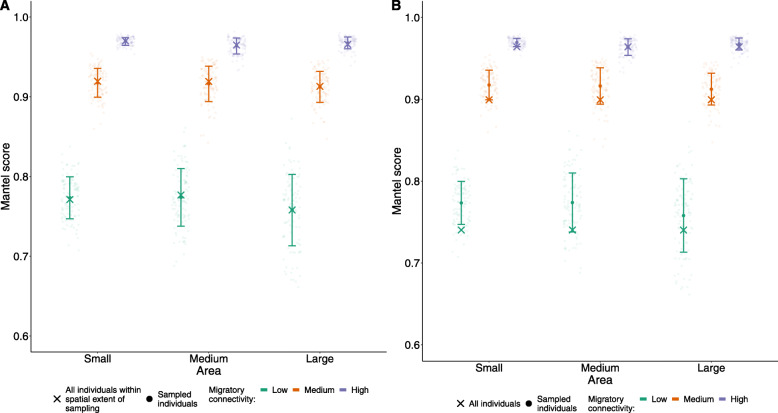
Mantel scores from 100 replicate simulated studies (circles) for each connectivity level, relative to ‘true’ values for all individuals within the zone of inference (crosses). **a** ‘True’ migratory connectivity value for the populations solely within the spatial zone of sampling. **b** ‘True’ migratory connectivity for a zone encompassing the entire population. Each simulated study comprised of 200 individuals sampled randomly across four equal sized sampling areas centred within sub-populations which varied in size. Error bars indicate standard deviation around the mean score of 100 replicates

In the scenario where random sampling was applied within increasingly-sized sampling zones across four distinct breeding sub-populations, inferences about the strength of connectivity for individuals inhabiting the strict zone of sampling produced metrics with limited bias (Fig. 5a). However, any attempt to extend inferences about the strength of migratory connectivity to the wider population beyond the spatial extent of sampling would lead to overestimation of migratory connectivity compared to the whole population (Fig. 5b). This bias was strongest in the scenario with smallest sampling area and weak connectivity.

### Complex patterns of bias emerge in more realistic population simulations

**Fig. 6:**
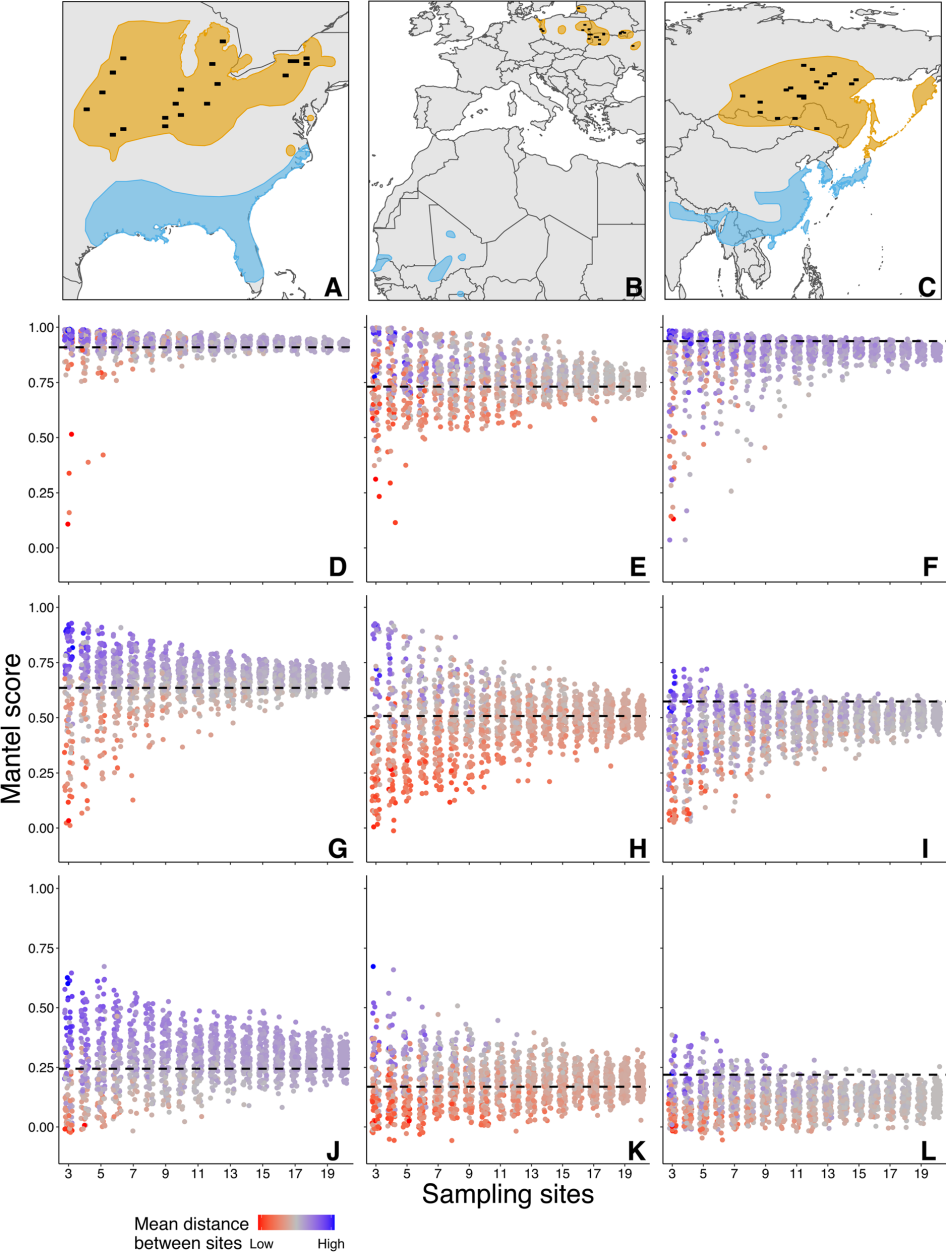
Breeding (orange) and non-breeding (light-blue) distributions of three simulated species (top, **a-c**), with filled black rectangles indicating the 20 potential sampling sites. Below each population are Mantel scores from replicate simulated studies applied to populations simulated within each range with three levels of connectivity; high (top, **d-f**), medium (middle, **g-i**), and low (bottom, **j-l**). Each point represents a sample of 200 individuals taken from between 3 and 20 sampling sites, with sites selected at random from the pools shown in Panels **a-c**. Point colour represents the mean distance between sampling sites in a given replicate study (blue is high, grey is medium, and red is low). Dashed horizontal line (**d**–**l**) indicates mantel score for the entire population

Applying realistic sampling regimes to real-world species range scenarios revealed how the mechanisms described above can lead to both over- and underestimation of true migratory connectivity estimates, depending on the spatial arrangement of sampled areas with respect to range geography. Across all three simulated ‘species’, bias was greatest when smaller numbers of discrete sampling locations were used (Fig. 6). With 5 or fewer sampling areas, replicate studies yielded hugely variable connectivity estimates within each population, with true connectivity tending to be underestimated when selected sites were relatively close together, and overestimated when they were far apart (Fig. 6).

The three simulated species showed subtly different patterns of overall bias, linked to the arrangement of potential sampled sites relative to the range as a whole. In the Henslow’s Sparrow range scenario, the sampled sites were widely dispersed across a large breeding range (Fig. 6a), leading to large between-site distances and hence consistent overestimation of connectivity, even when large numbers of sites were used (Fig. 6d, g, and j). In the Falcated Duck range scenario, all sampled sites fell within a more discrete area within the range (Fig. 6c), resulting in systematic underestimation of population-scale connectivity, regardless of the number of sampling sites used (Fig. 6f, i, and l). Where the spatial extent of sampling provided consistent coverage of the entire population, as with the Aquatic warbler range scenario (Fig. 6b), there was no consistent pattern of directional bias, but estimates remained hugely variable and often yielded large over- and underestimates of true connectivity depending on the precise arrangement of sites selected from the pool available (Fig. 6e, h, and k).

## Discussion

There is an increasing demand in ecology and conservation for robust measures of the strength of migratory connectivity [5]. We have shown the potential for bias and high uncertainty when broad-scale inferences about connectivity are made using Mantel statistics, driven by the fundamental dependence of these metrics on the spatial arrangement of samples. The strength of connectivity in a single population, as measured by the Mantel method, will always vary with the spatial scale of inference, with larger spatial sub-sections of a population likely to show higher connectivity scores. As a result, any attempt to infer the strength of connectivity for a contiguous species-level distribution from a spatial subset of individuals may lead to biased estimates. If inference is restricted to the strict spatial extent of sampling, random sampling across the area of inference can produce unbiased estimates of connectivity, but the use of disparate sampling sites is likely to cause overestimation. Even under ideal sampling practices (random sampling across a single area), which are in many cases unachievable, replicate estimates for a single population vary widely when sample sizes are small (100 or fewer tracked individuals). Within more complex and realistic simulated populations, these mechanisms can generate huge variation in connectivity estimates derived for a single population, depending on exactly where within the range samples are taken. These results indicate strong dependence of Mantel metrics on the design of spatial sampling regimes, and can help to inform best-practice in study design and improve accuracy.

Previous studies have suggested combining the Mantel test with cluster analysis to control for population structuring [11], as well as calls for the measure to be used in conjunction with an absolute measure of population spread (the degree to which individuals from a single breeding population spread out during the non-breeding season) to better disentangle the properties of connectivity [18]. These proposals do not, however, address fundamental biases that arise from constraints on observable pairwise distances under different sampling regimes, demonstrated by our simulations. Whilst recently-developed extensions to the Mantel approach using between-region transition rates could plausibly account for some of these biases [12], the data requirements for these methods can be prohibitive, and use of Mantel statistics remains widespread (e.g. [13–16], but see [9, 17]). Our simulations suggest that biases can only be avoided by careful study design, and in particular the explicit restriction of inference to clearly delimited spatial subsets of populations.

Our results suggest that making inferences about the strength of connectivity for wider populations beyond the spatial extent of sampling can lead to significant bias (Figs. 2 and 5). As metrics of connectivity are fundamentally scale dependent, ‘true’ connectivity values for sub-populations within a small portion of the breeding range are likely to be lower than ‘true’ values calculated for the entire breeding range, even if movement behaviour is universally governed by the same process. This illustrates that connectivity, as measured by these metrics, is a spatial pattern and not necessarily a fundamental species-level trait or characteristic.

Given that real-world studies may be limited to sampling individuals from small subsets of their target species’ ranges, our results suggest that underestimation of population-scale migratory connectivity is likely to be commonplace. However, in species with spatial heterogeneity in migratory programme such as migratory divides (e.g. Barn Swallow *Hirundo rustica* in North America [29];), the directionality of this scale-dependent bias may be further complicated by what sub-population is sampled. Accurate estimation of connectivity at a population level may therefore only be possible where sampling is exhaustive, but even here researchers must carefully consider their sampling design. True random sampling is likely to be impossible to achieve across large areas, especially with study species that are difficult to trap, or tag retrieval is required. A multiple sampling site approach may be more feasible to implement in practice (e.g. breeding site sampling locations in [18]), yet the sites should be well distributed throughout the area of inference, with careful consideration of site spacing. Sites either spaced very far apart, or clumped close together, may lead to biased metrics with respect to wider populations. Our more realistic simulated scenarios suggest that more accurate estimates of connectivity will be achieved by using larger numbers of sampling sites spread across the area of inference, even if this leads to lower sample sizes of marked individuals within each site. Practitioners must therefore balance maximising the number of sampling sites used, with the practical limitations of such study designs.

Whilst our results are limited to simulations, the scenarios we have examined can be considered as simplified ‘best-case’ scenarios for sampling regimes. Real-world data collection will inevitably be more complex, and may include additional factors of bias such as non-representative sampling of a population and differential survival rates between cohorts that may affect tag retrieval [30]. In practice, ascertaining the connectivity of an entire large population using Mantel correlations may always be unfeasible, and in these cases, care should be taken to avoid making general population-level inferences from subsamples. Given the potential for bias, we suggest that researchers should carefully consider on a case-by-case basis whether simpler visual representations of spatial connectivity may ultimately be just as informative as quantitative connectivity metrics. In some use cases, such as temporal comparisons of Mantel statistics within a single study [31], biases may be consistent with respect to the variable of interest (time) and thus allow for robust comparisons. Comparisons of Mantel scores between studies, however, are likely to be particularly vulnerable to bias, especially where sampling regimes differ substantially.

## Conclusions

Due to fundamental scale-dependence, the notion of a single ‘true’ connectivity value that applies to a species is unlikely to be realistic. The development of new and broadly-applicable statistical methods to control for this spatial dependence would be extremely valuable for future connectivity research. Nevertheless, our work suggests that with good sampling design and explicit clarity over the spatial extent where inference is made, deriving meaningful population-level measures of connectivity using Mantel correlations remains feasible. Where random sampling of individuals across the whole area of inference is not possible, we recommend maximising the number of discrete sampling sites and avoid cases where sampling site spacing (either too much or too little) is likely to cause overt bias. We also strongly advise against making inferences about the strength of connectivity of a population that extends well beyond the spatial extent of sampling. We hope that with these recommendations, measures of connectivity will be more robust.

## Supplementary Information

**Additional file 1: Figure A1.** Simulating the breeding and non-breeding locations of 10,000 individuals. A. An individual is given a breeding location by placing the individual at random within the breeding area. The individual is then moved a set distance in a southerly direction B. The individual is then moved to a final non-breeding location. The direction of this movement is taken at random (C *left*) and the distance is drawn from a log-normal distribution (C *right*) which we varied to change the relative strength of migratory connectivity. D. This process is repeated for 10,000 individuals in the simulated population.

**Additional file 2: Figure A2.** Hypothetical examples showing the spatial distribution (A) of breeding (yellow dots) and non-breeding locations (blue dots) for a migratory patchy population and the corresponding frequency distributions (B) of pairwise distances between individuals during breeding (yellow line) and non-breeding (blue line). Panels C, E and G show increasingly large spatial subsections within each sub-population, together with the corresponding pairwise distance frequency distributions (D, F and H), highlighting how distance distributions vary with sampling area for breeding, but less so in non-breeding seasons. Total population is shown as translucent and individuals within a spatial subsection shown coloured in in plots C, E and G.

**Additional file 3: Figure A3.** Production and sampling of a simulated realistic migratory population. A) Generating spatially autocorrelated occurrence probability values across a real-world species range (breeding range Falcated Duck *Mareca falcata* shown here). B) 50,000 individuals are then distributed across the range with locations weighted by cell occurrence probabilities (10,000 shown here). C) Individuals are then linked between seasons by random selection of corresponding points, varying the level of simulated connectivity by changing the bandwidth of longitudinal rank (1000 individuals shown here, low-medium migratory connectivity scenario). D) A coarse grid is overlaid on the breeding zone, across which we calculate the number of individuals in each cell. E) The 20 cells with the highest number of individuals are taken as sampling sites.

**Additional file 4: Figure A4.** Mantel scores of sampled individuals (circles) compared to ‘true’ values for the whole population (crosses) across three simulated connectivity levels. Each replicate is calculated using 200 individuals sampled randomly across a single area which was varied in size. Error bars indicate standard deviation around the mean score of 100 replicates.

**Additional file 5: Figure A5.** Mantel scores from 100 replicate simulated studies (circles) compared to that of all individuals within the spatial extent of sampling (crosses). Samples comprised of 200 individuals sampled randomly across a single area which was changed in size. Error bars indicate standard deviation around the mean score of 100 replicates.

**Additional file 6: Figure A6.** A-C: Density plots of pairwise distances between individuals under spread-based scenarios, depicting how the distribution of sampled pairwise distances (solid lines) varies with the scale of sampling, relative to true distance distributions for the whole population (dotted lines). Inset schematics visualise the sampling regime on the breeding ground, with highlighted region indicating the zone of sampling. D: Mantel scores from 100 replicate simulated studies (circles), compared to the whole population of 10,000 individuals (crosses). Samples comprised 200 individuals chosen randomly across the nine sampling areas which varied in their spread. Error bars indicate standard deviation around the mean score of 100 replicates. These examples show the lowest level migratory connectivity simulated (Mantel MC 0.33).

## Data Availability

The code and data used to conduct this analysis can be accessed at www.github.com/SHVickers94/Sensitivity-of-migratory-connectivity-metrics-to-spatial-sampling-design (Vickers, 2021).
