# Supplementary figures and images for: Sensitivity of migratory connectivity estimates to spatial sampling design
Source: Mov Ecol. 2021 Apr 2;9:16. doi: 10.1186/s40462-021-00254-w (PMC8019184; doi:10.1186/s40462-021-00254-w)

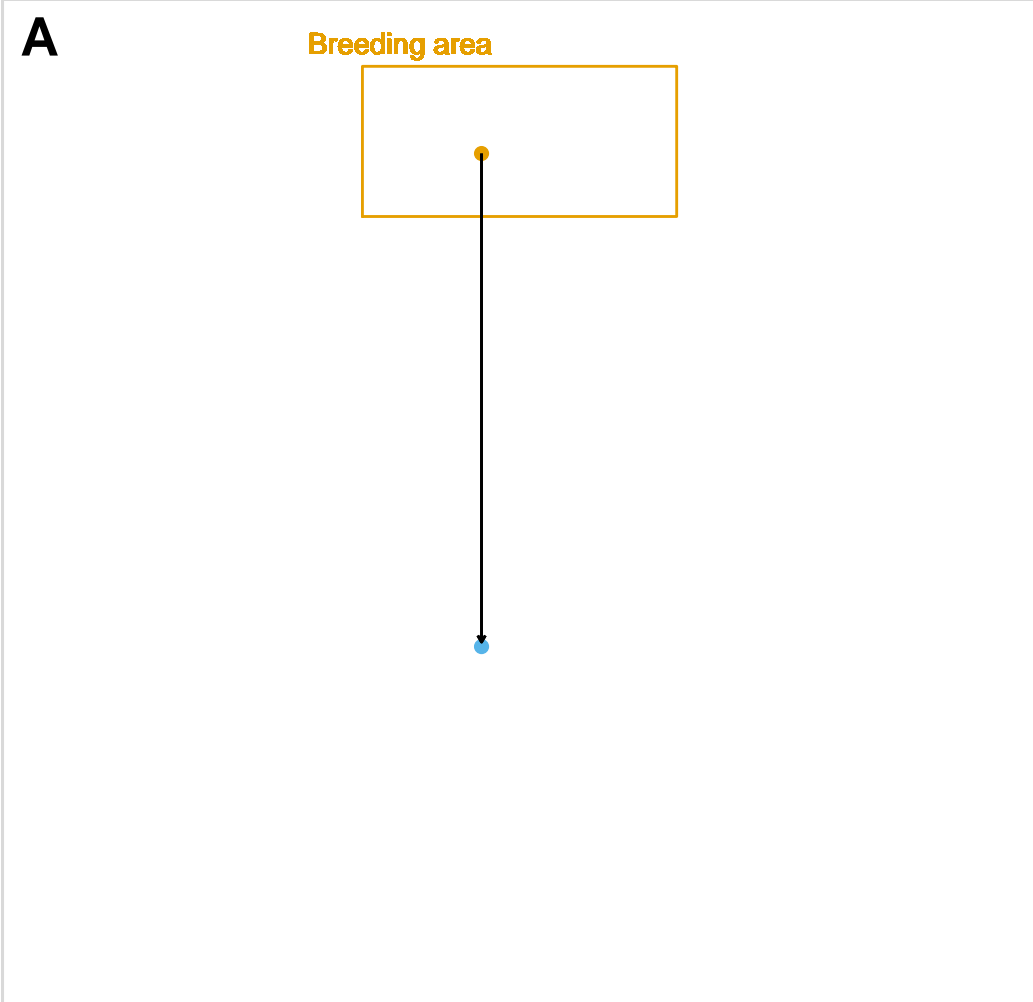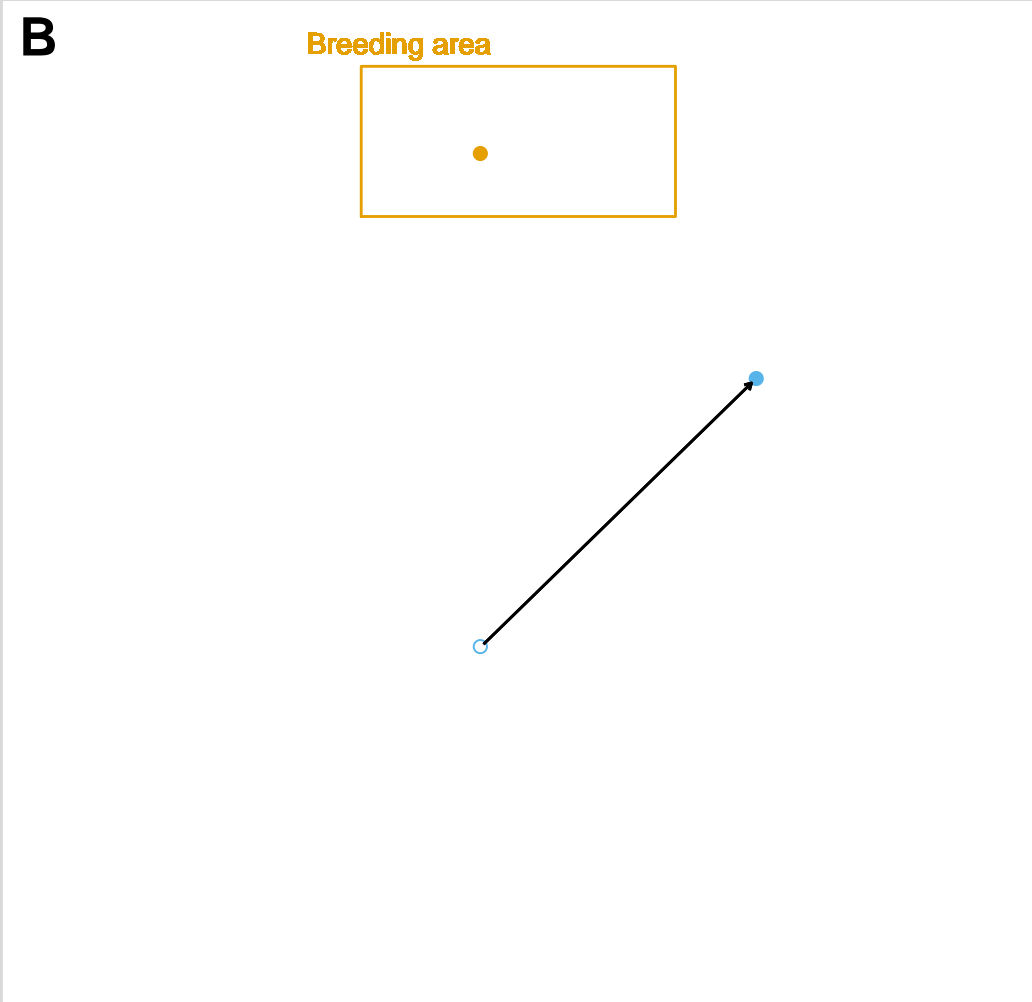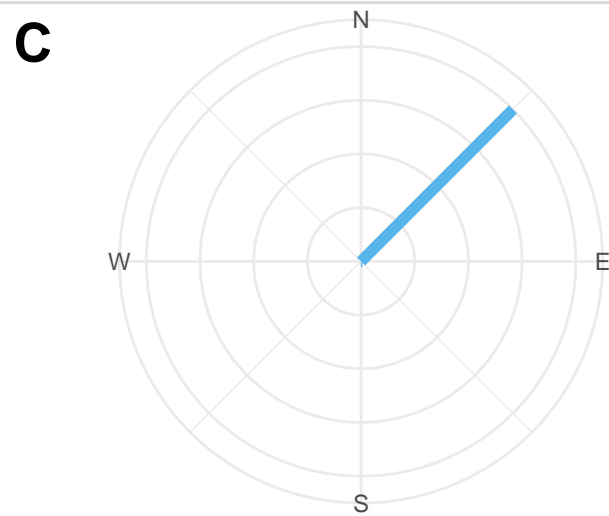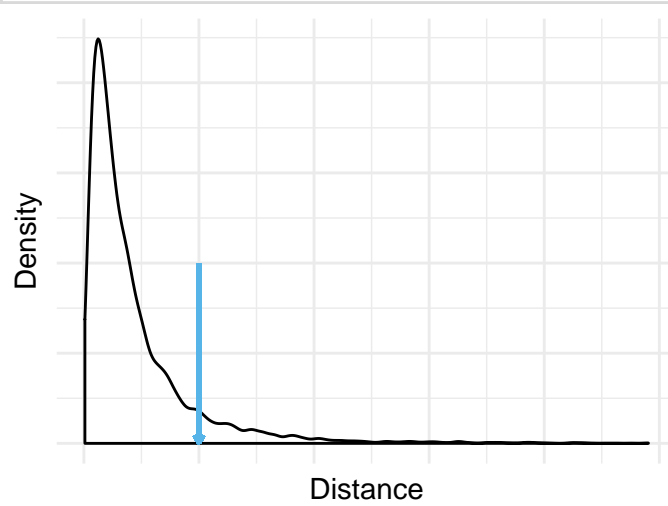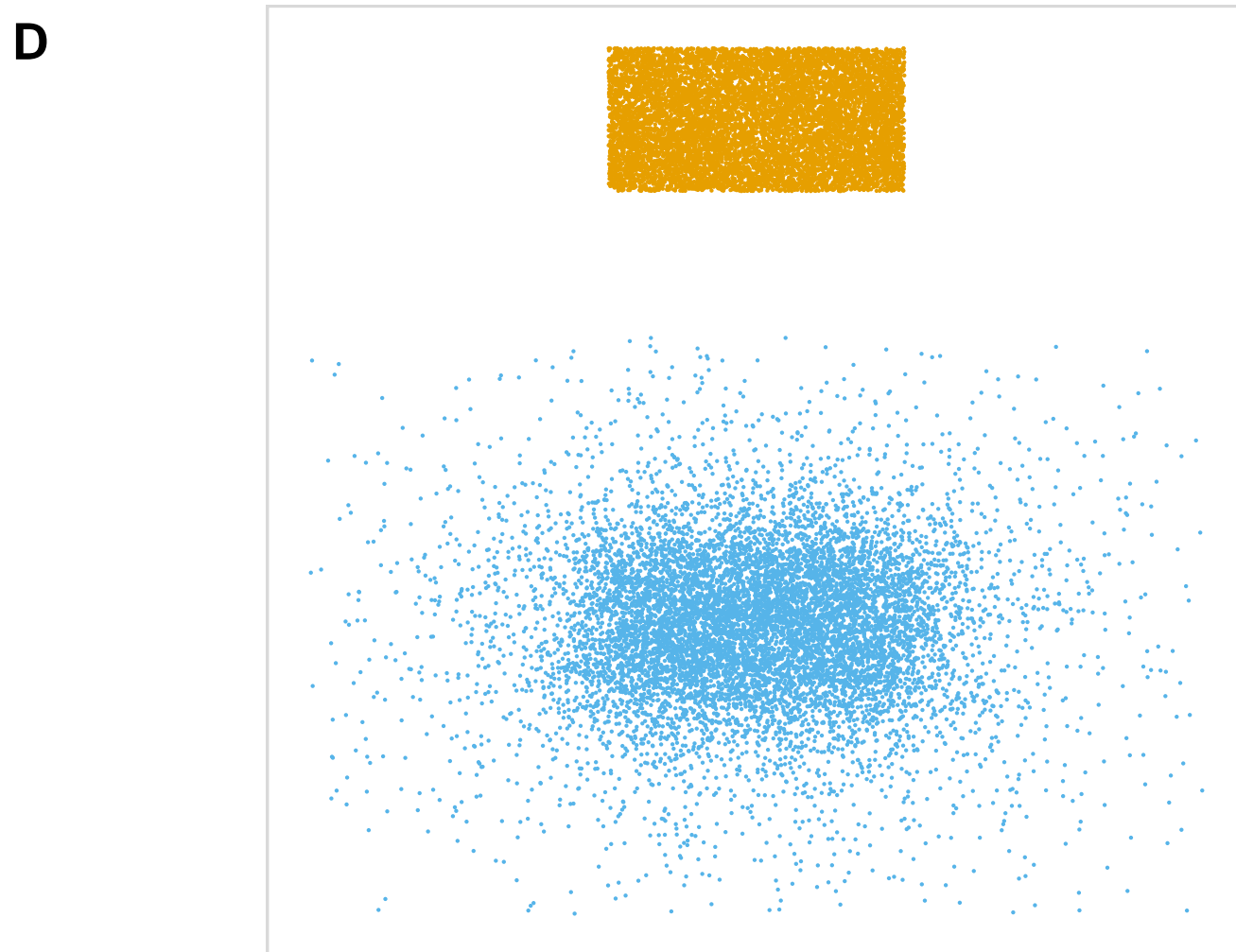

Supplement: Supplementary file 1 — Additional file 1: Figure A1. Simulating the breeding and non-breeding locations of 10,000 individuals. A. An individual is given a breeding location by placing the individual at random within the breeding area. The individual is then moved a set distance in a southerly direction B. The individual is then moved to a final non-breeding location. The direction of this movement is taken at random (C left) and the distance is drawn from a log-normal distribution (C right) which we varied to change the relative strength of migratory connectivity. D. This process is repeated for 10,000 individuals in the simulated population. [file 40462_2021_254_MOESM1_ESM.pdf]

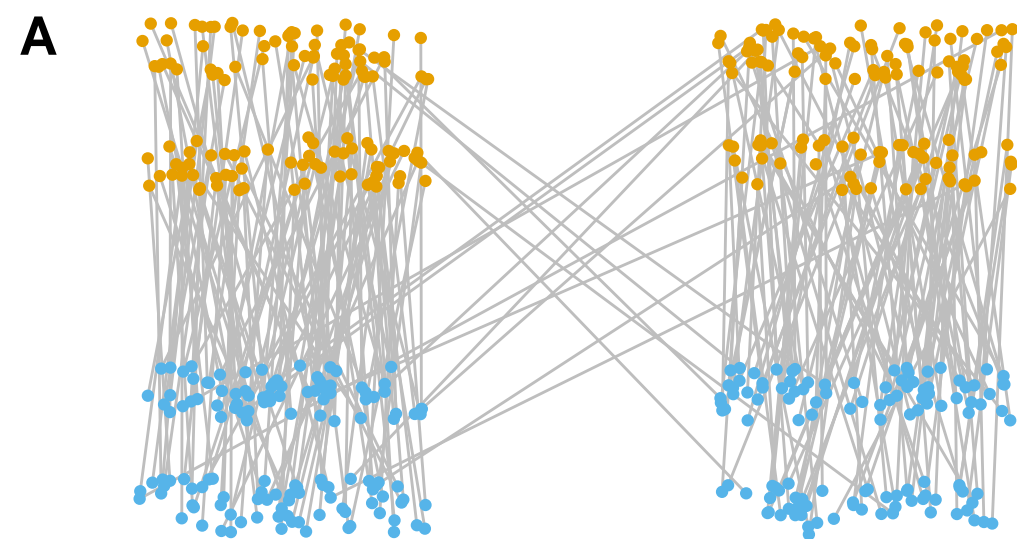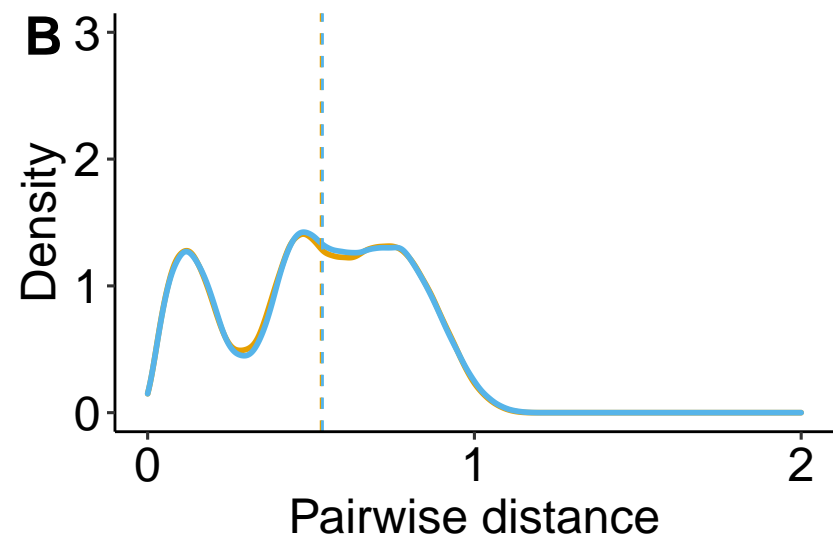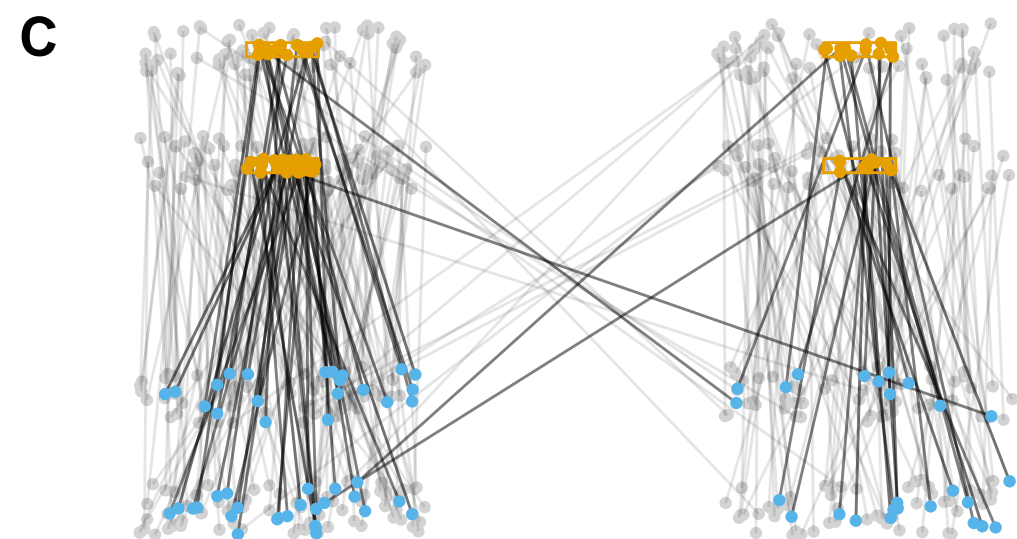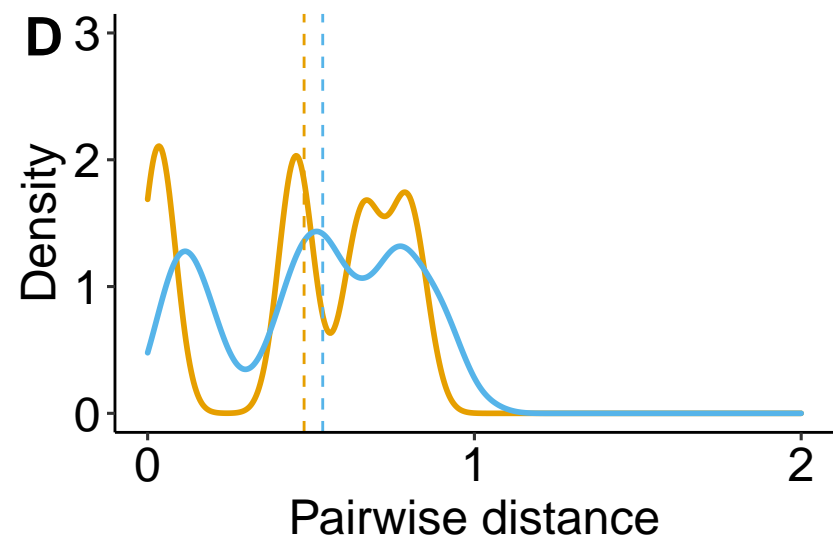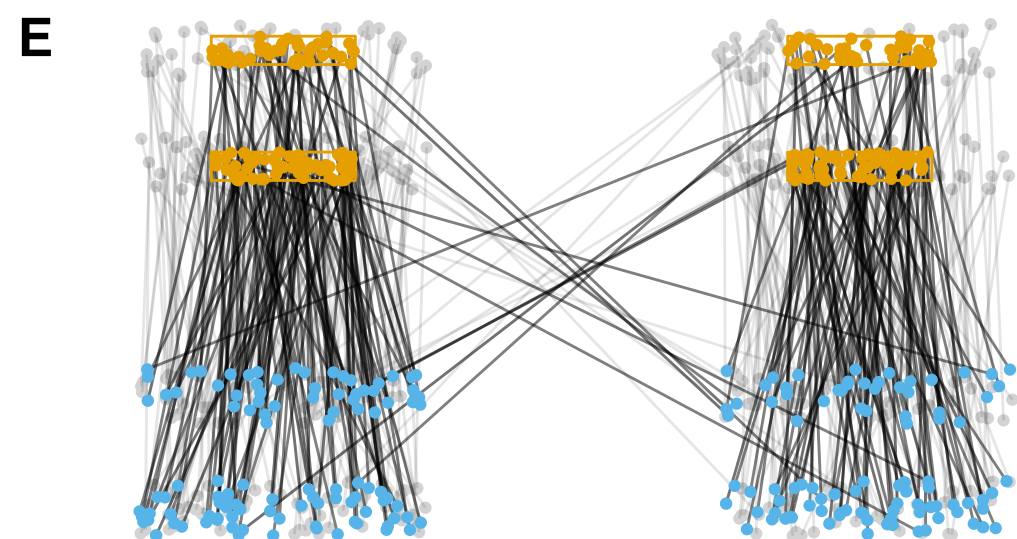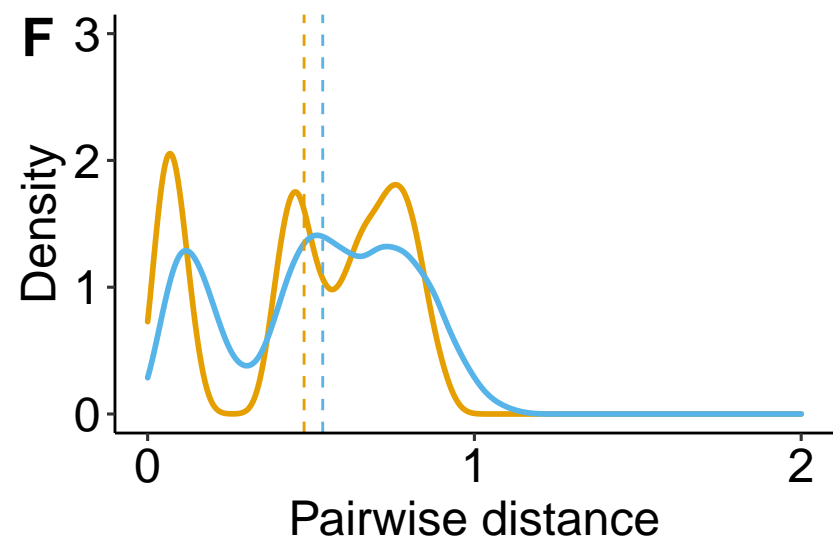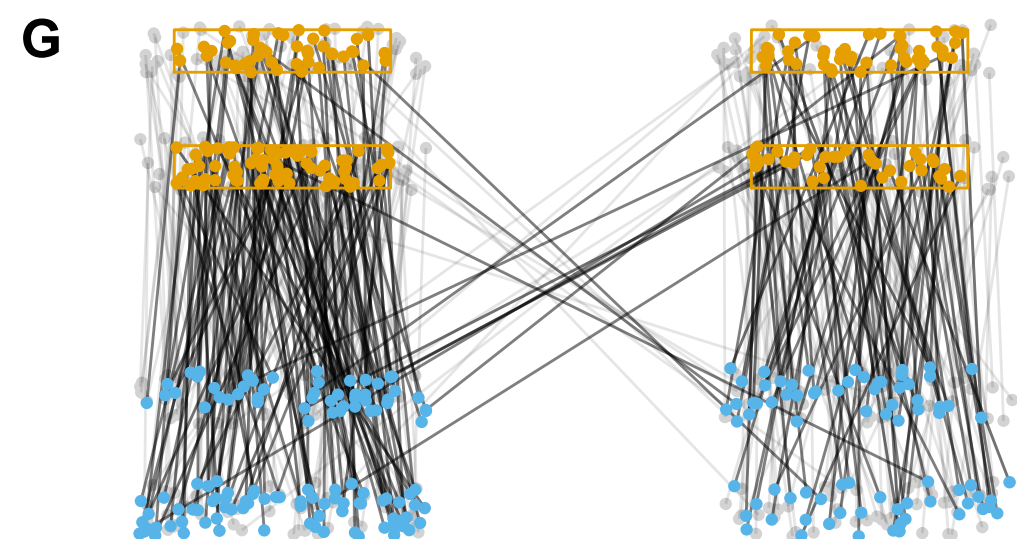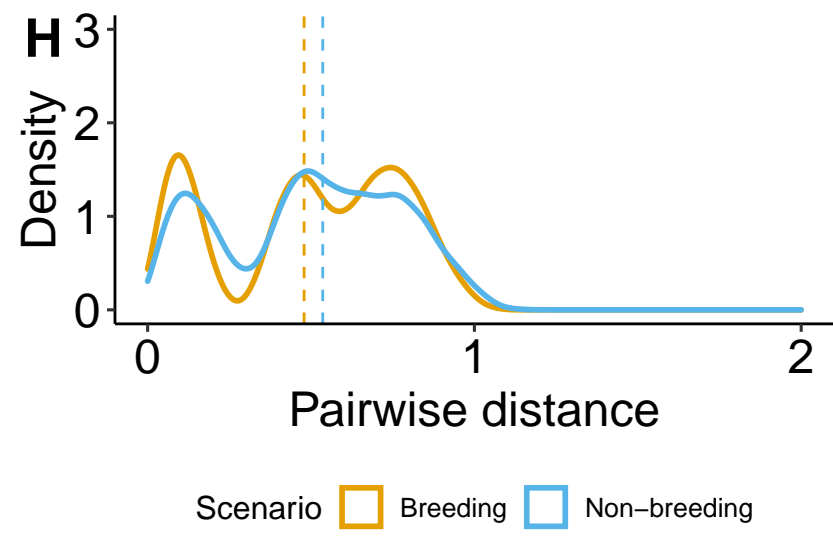

Supplement: Supplementary file 2 — Additional file 2: Figure A2. Hypothetical examples showing the spatial distribution (A) of breeding (yellow dots) and non-breeding locations (blue dots) for a migratory patchy population and the corresponding frequency distributions (B) of pairwise distances between individuals during breeding (yellow line) and non-breeding (blue line). Panels C, E and G show increasingly large spatial subsections within each sub-population, together with the corresponding pairwise distance frequency distributions (D, F and H), highlighting how distance distributions vary with sampling area for breeding, but less so in non-breeding seasons. Total population is shown as translucent and individuals within a spatial subsection shown coloured in in plots C, E and G. [file 40462_2021_254_MOESM2_ESM.pdf]

**A**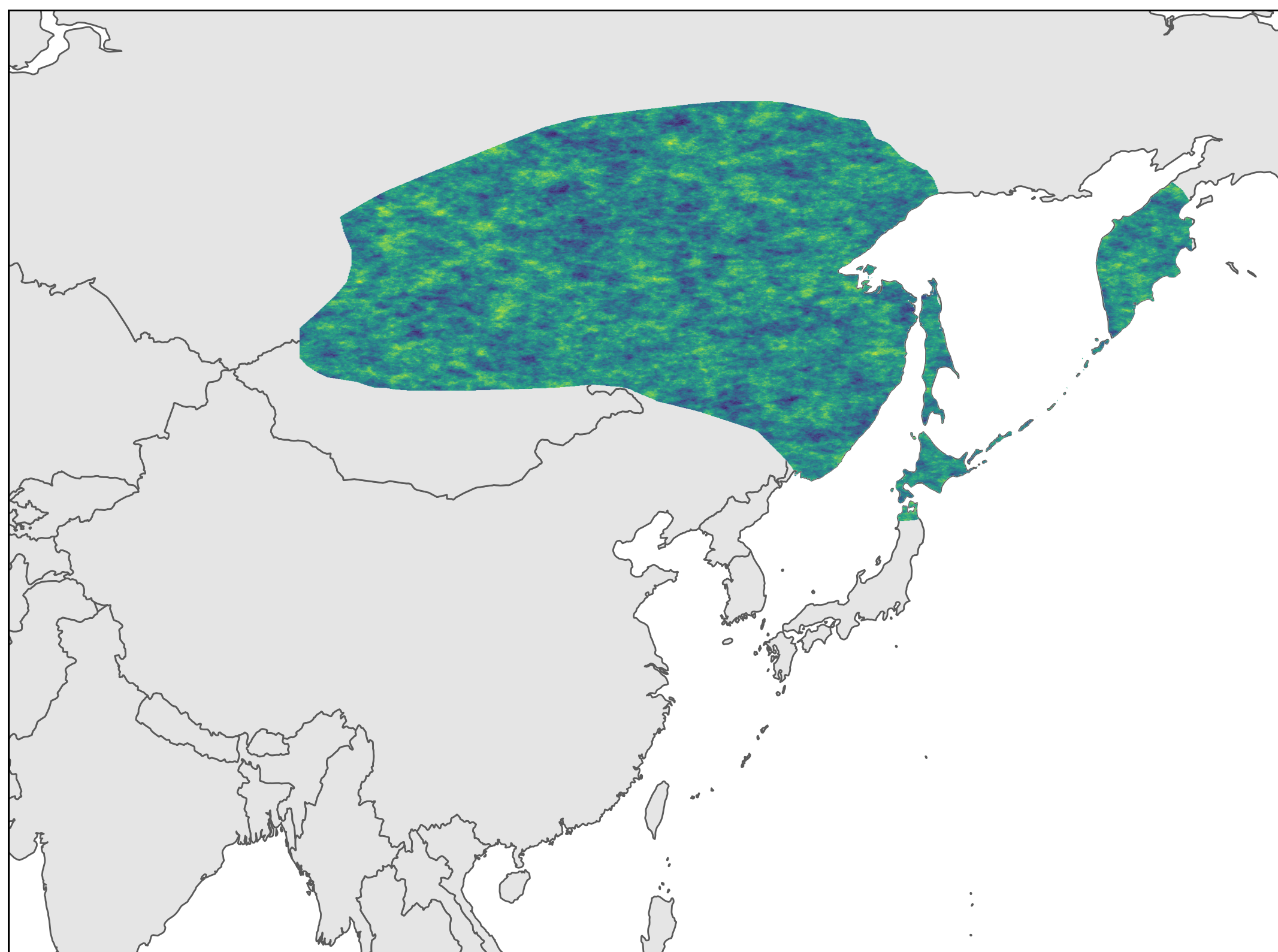**B**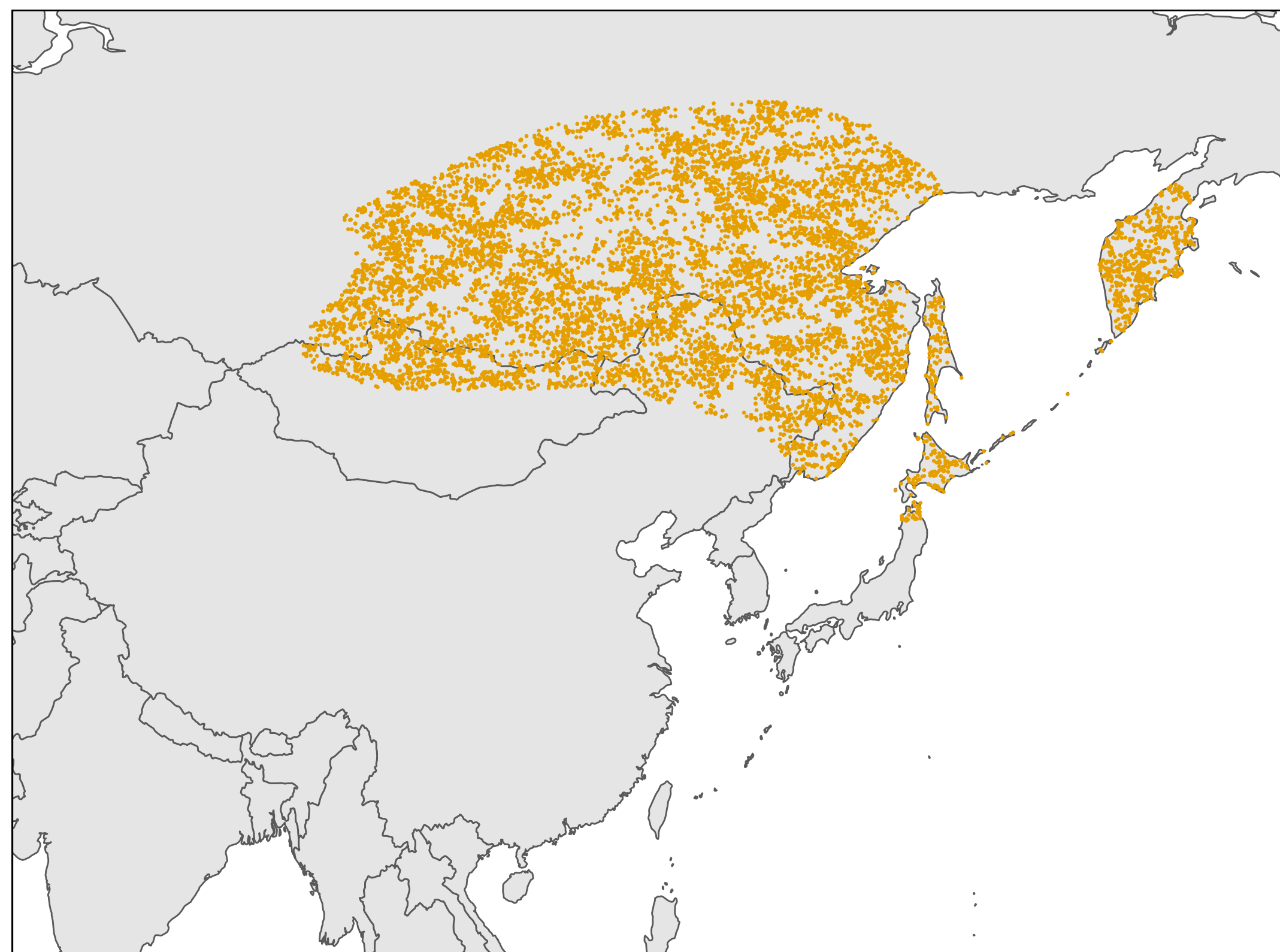**C**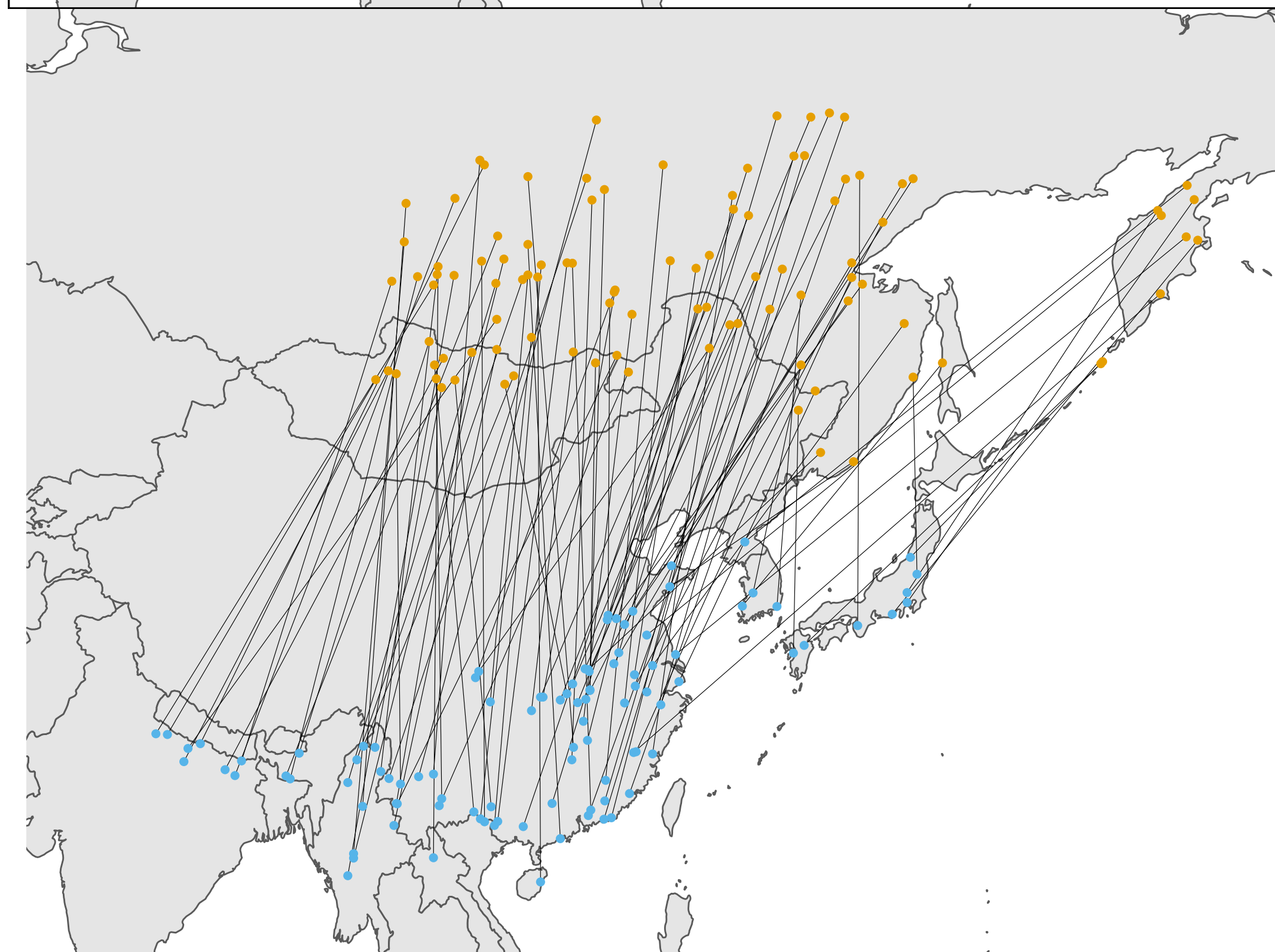**D**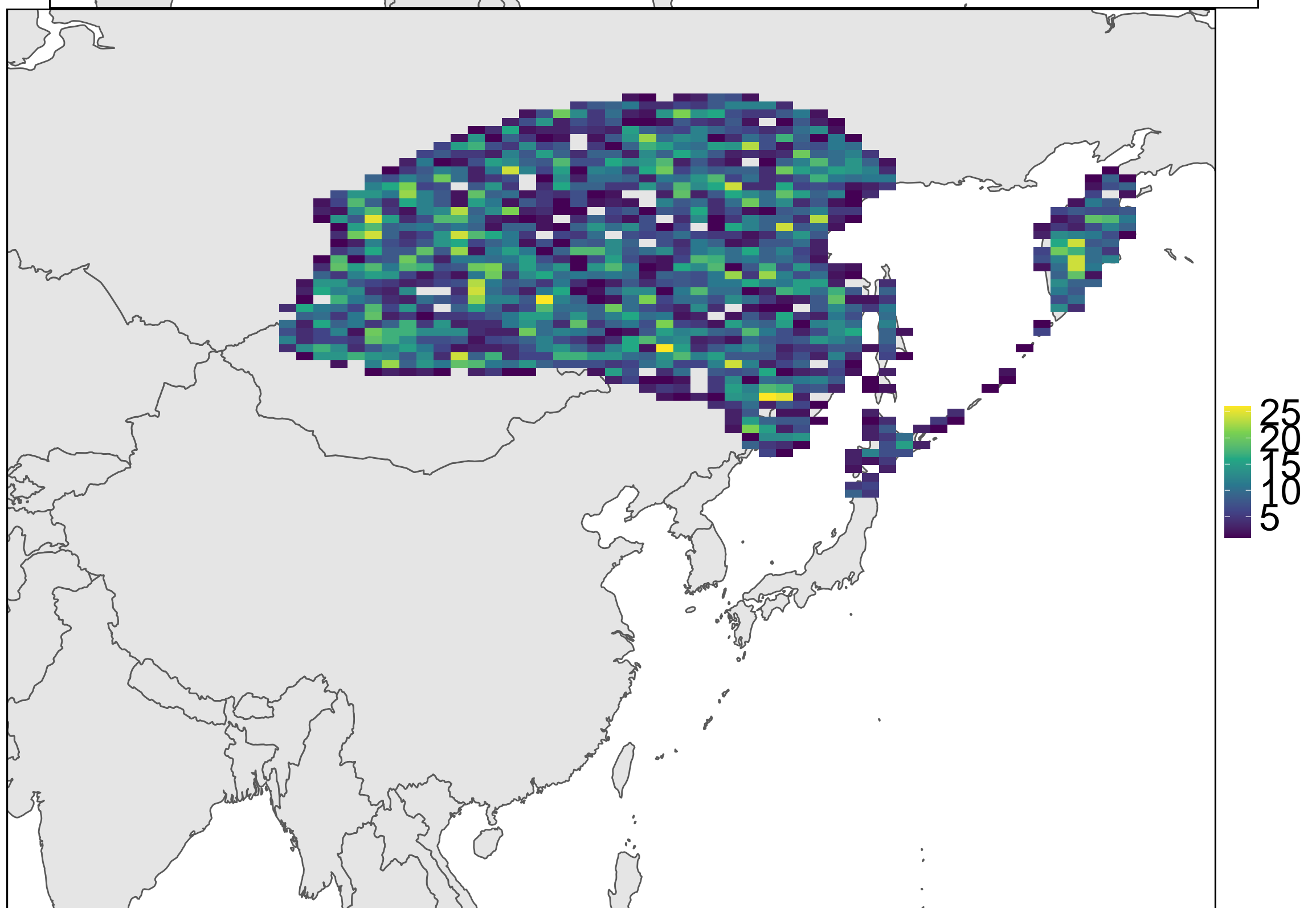**E**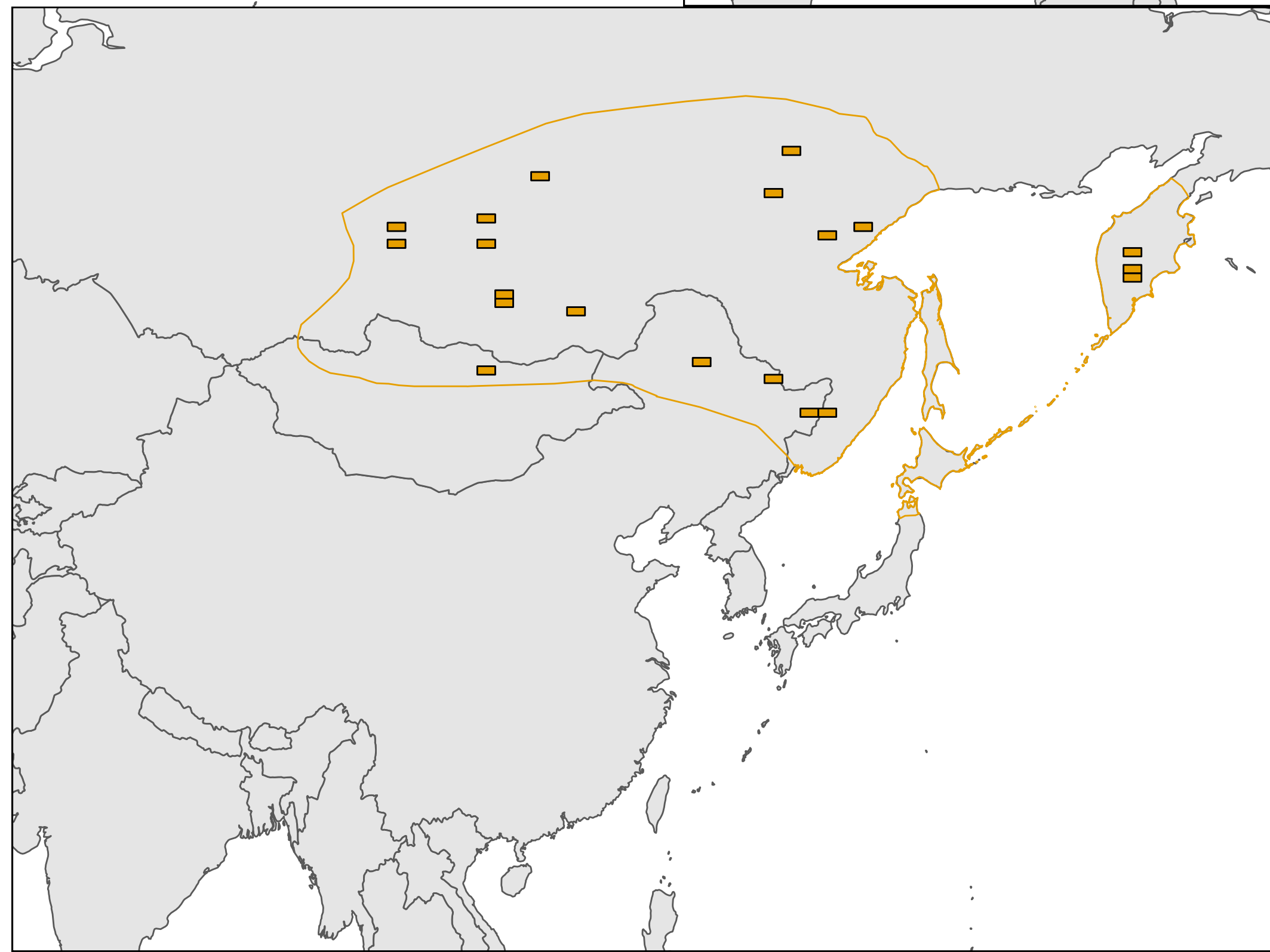

Supplement: Supplementary file 3 — Additional file 3: Figure A3. Production and sampling of a simulated realistic migratory population. A) Generating spatially autocorrelated occurrence probability values across a real-world species range (breeding range Falcated Duck Mareca falcata shown here). B) 50,000 individuals are then distributed across the range with locations weighted by cell occurrence probabilities (10,000 shown here). C) Individuals are then linked between seasons by random selection of corresponding points, varying the level of simulated connectivity by changing the bandwidth of longitudinal rank (1000 individuals shown here, low-medium migratory connectivity scenario). D) A coarse grid is overlaid on the breeding zone, across which we calculate the number of individuals in each cell. E) The 20 cells with the highest number of individuals are taken as sampling sites. [file 40462_2021_254_MOESM3_ESM.pdf]

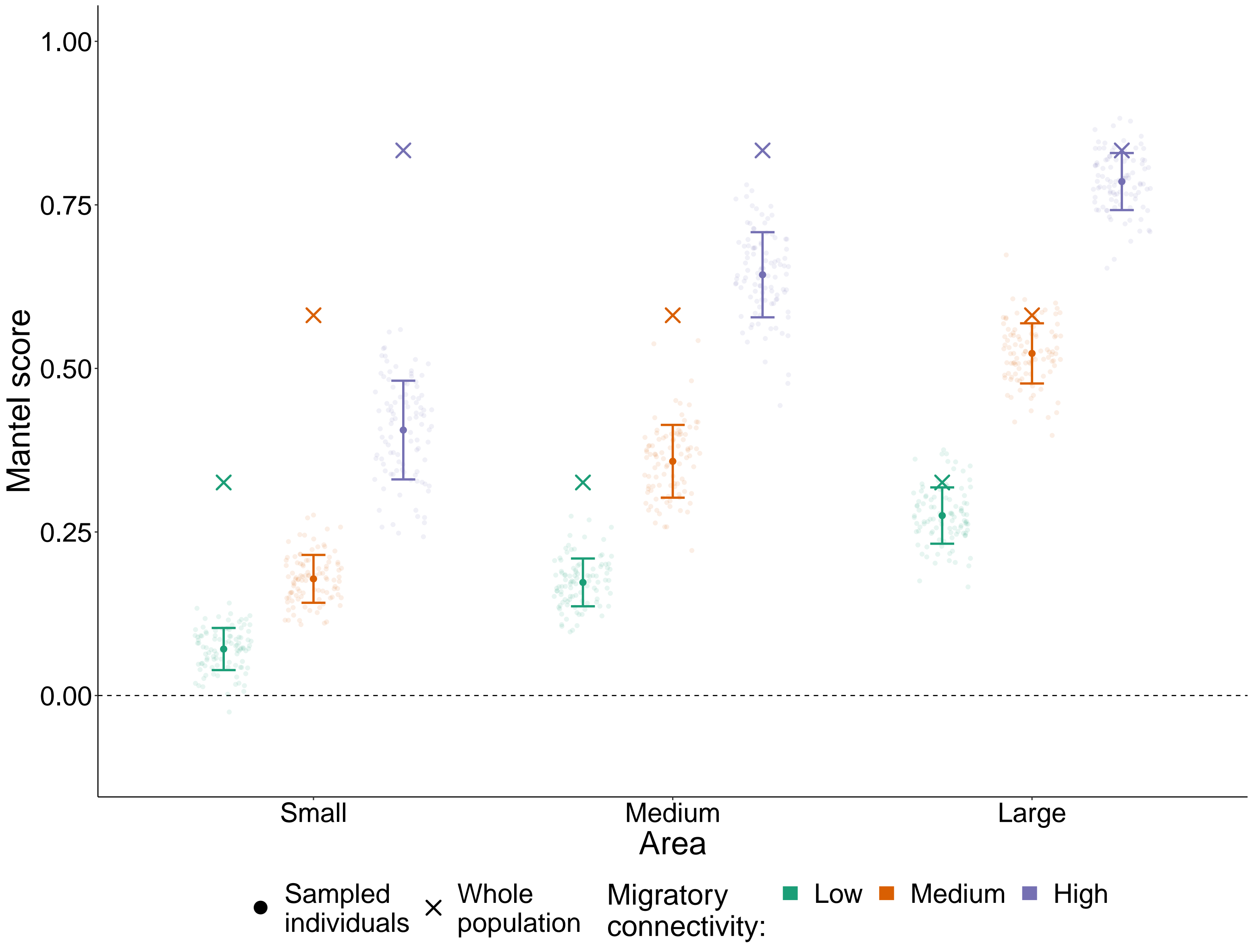

Supplement: Supplementary file 4 — Additional file 4: Figure A4. Mantel scores of sampled individuals (circles) compared to ‘true’ values for the whole population (crosses) across three simulated connectivity levels. Each replicate is calculated using 200 individuals sampled randomly across a single area which was varied in size. Error bars indicate standard deviation around the mean score of 100 replicates. [file 40462_2021_254_MOESM4_ESM.pdf]

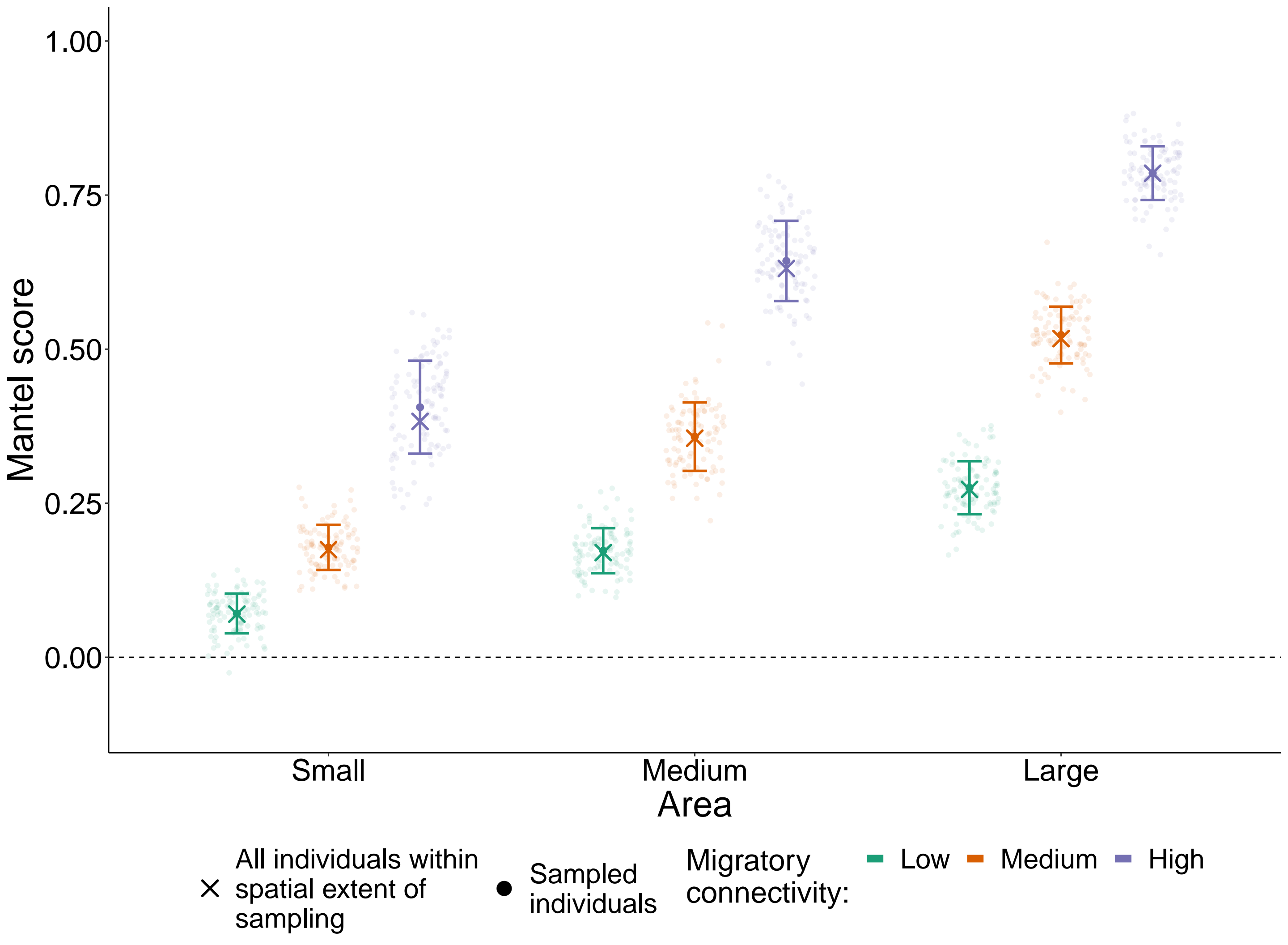

Supplement: Supplementary file 5 — Additional file 5: Figure A5. Mantel scores from 100 replicate simulated studies (circles) compared to that of all individuals within the spatial extent of sampling (crosses). Samples comprised of 200 individuals sampled randomly across a single area which was changed in size. Error bars indicate standard deviation around the mean score of 100 replicates. [file 40462_2021_254_MOESM5_ESM.pdf]

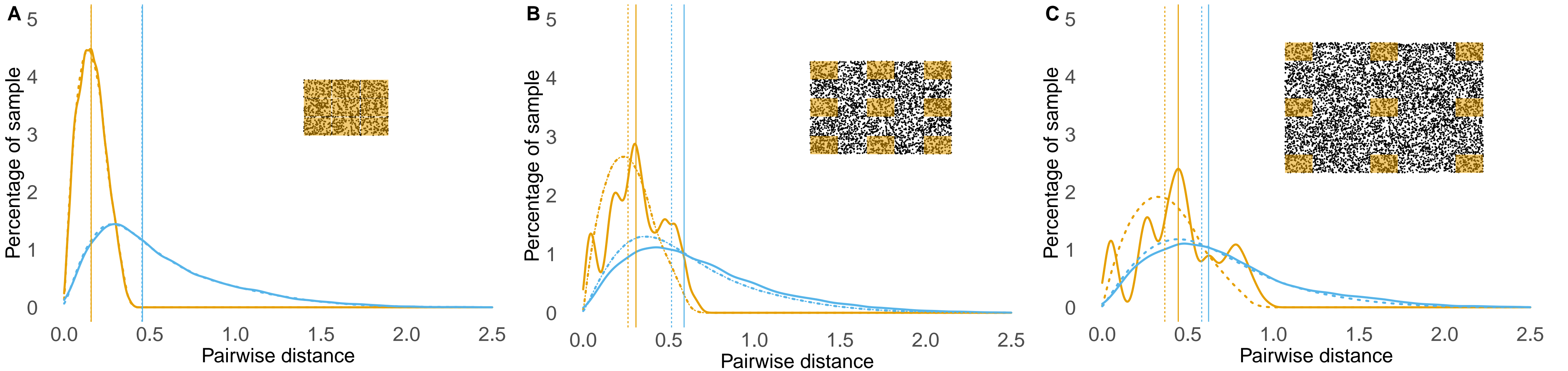

**Sample** + Sampled individuals + Whole population **Scenario** + Breeding + Non-breeding

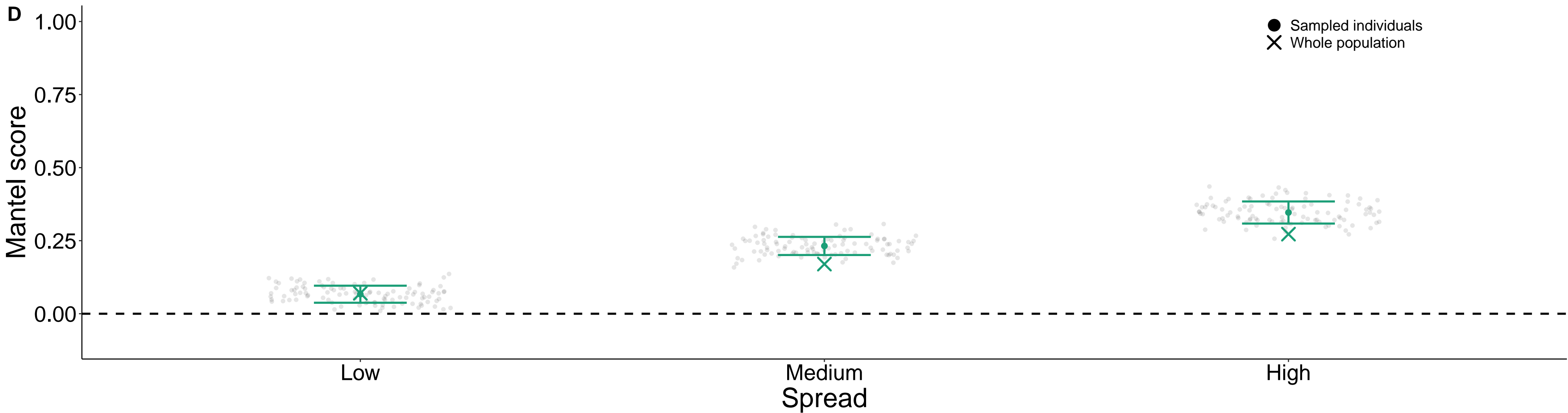

Supplement: Supplementary file 6 — Additional file 6: Figure A6. A-C: Density plots of pairwise distances between individuals under spread-based scenarios, depicting how the distribution of sampled pairwise distances (solid lines) varies with the scale of sampling, relative to true distance distributions for the whole population (dotted lines). Inset schematics visualise the sampling regime on the breeding ground, with highlighted region indicating the zone of sampling. D: Mantel scores from 100 replicate simulated studies (circles), compared to the whole population of 10,000 individuals (crosses). Samples comprised 200 individuals chosen randomly across the nine sampling areas which varied in their spread. Error bars indicate standard deviation around the mean score of 100 replicates. These examples show the lowest level migratory connectivity simulated (Mantel MC 0.33). [file 40462_2021_254_MOESM6_ESM.pdf]
